# Modeling Misinformation Spread in a Bounded Confidence Model: A Simulation Study

**DOI:** 10.3390/e26020099

**Published:** 2024-01-23

**Authors:** Yujia Wu, Peng Guo

**Affiliations:** School of Management, Northwestern Polytechnical University, Xi’an 710021, China; guopeng@nwpu.edu.cn

**Keywords:** misinformation, bounded confidence, opinion dynamics, small-world networks, heterogeneity

## Abstract

Misinformation has posed significant threats to all aspects of people’s lives. One of the most active areas of research in misinformation examines how individuals are misinformed. In this paper, we study how and to what extent agents are misinformed in an extended bounded confidence model, which consists of three parts: (i) online selective neighbors whose opinions differ from their own but not by more than a certain confidence level; (ii) offline neighbors, in a Watts–Strogatz small-world network, whom an agent has to communicate with even though their opinions are far different from their own; and (iii) a Bayesian analysis. Furthermore, we introduce two types of epistemically irresponsible agents: agents who hide their honest opinions and focus on disseminating misinformation and agents who ignore the messages received and follow the crowd mindlessly. Simulations show that, in an environment with only online selective neighbors, the misinforming is more successful with broader confidence intervals. Having offline neighbors contributes to being cautious of misinformation, while employing a Bayesian analysis helps in discovering the truth. Moreover, the agents who are only willing to listen to the majority, regardless of the truth, unwittingly help to bring about the success of misinformation attempts, and they themselves are, of course, misled to a greater extent.

## 1. Introduction

Misinformation has become an inextricable part of the modern world, and the widespread dissemination of which has posed significant threats to all areas of people’s lives. During the COVID-19 pandemic, the exposure to misinformation prompted engagement in misinformed behaviors and discouraged evidence-based prevention behaviors [1,2]. Misinformation about climate change confuses the public, leads to political inaction, and leads to the rejection of mitigation policies [3].

The spread of misinformation on social networks has been the central focus of some recent work. Some studies consider binary opinion dynamics. For example, the topic might be believing or not believing a certain message. Acemoglu and Mostagir considered whether agents will effectively weed out incorrect beliefs according to dispersed information and thus learn the truth [4,5]. Nguyen et al. focused on how many agents will be influenced by misinformation to take incorrect actions and how to minimize the number of those agents [6,7,8]. Besides taking two extremes (true or false), one can envision a continuous opinion space but the actions taken are discrete [9,10,11]. Liu et al. inferred the diffusion rate of misinformation and whether agents can debunk misinformation by calculating the densities of users with different opinions [12,13].

Unlike the limited options presented in those papers, the opinions can sometimes be represented by continuous variables. A particular type of agent holds an opinion that can be any real number in the interval [0, 1], for example, 0.8. Researchers run simulations to determine the average number of agents that started with different initial opinions and have been converted to a fixed point (i.e., 0.8). Most of this work assumes that the social network consists of strictly sincere agents, all willing to tell what they really think when communicating with others [14,15,16,17]. Douven and Hegselmann considered the possibility that some agents may have ulterior motives, including carrying out misinformation campaigns [18]. In this study, the agents’ opinions are represented by continuous variables in the interval [0, 1], where zero and one illustrate the misinformation and the truth, respectively. We investigated how individuals are misled to the misinformation side.

Much of the literature studying continuous opinions focuses on the bounded confidence model in which an agent only interacts with those whose opinions differ from their own by less than a certain value. Two well-known bounded confidence models were introduced by Hegselmann and Krause (HK) [19,20,21] and Deffuant, Weisbuch et al. (DW) [22,23]. These two models differ in the communication regime: in the DW model, agents meet in random pairwise encounters and change their opinions to the average of both opinions if they are within each other’s confidence intervals; in the HK model, the most well-known synchronous version of the DW model, each agent takes the arithmetic mean of opinions within their confidence interval, and all agents update at the same time.

There are many extensions of the bounded confidence model in different respects. Some works assume situations where all individuals have the same level of confidence [24,25,26]. However, more extensions assume that the agents’ bounds of confidence are heterogeneous. Lorenz proposed a society of agents with two different bounds of confidence (open-minded with a broader confidence interval and closed-minded with a narrower one) [27,28]. The confidence radii in some works are even agent-specific [29,30,31,32,33,34] or time-varying [18,35]. Furthermore, Hegselmann et al. considered asymmetric confidence intervals, where an agent took a broader range of opinions on their left (right) than on their right (left) into consideration if we represented the opinions on an x-axis [15,21,26,35]. Some researchers also considered agents who never update their opinions and examine their impact on other normal individuals and the trend of public opinion in social networks. Some papers refer to such agents as opinion leaders [36,37], others as mass media [33,38], and a few as stubborn agents [39,40].

Inspired by those studies, we present an extended BC model with “stubborn agents” and heterogeneous bounds of confidence. What makes our model different from the previous ones is that the agents adopt different confidence intervals for different kinds of neighbors. Knop et al. compared online and offline communication and argue that, though people exchange messages more often online, face-to-face communication leads to a higher amount and more depth of self-disclosure [41]. Thus, we bring not only online but also offline opinion dynamics into the model, where the agents act open-minded when facing their offline neighbors but are relatively closed-minded when interacting with their online selective neighbors. Furthermore, “stubborn agents” appear in the form of “paid posters”, a type of epistemically irresponsible agent, who deliberately disseminate misinformation and keep their opinions unchanged at “0”. An interesting and practical assumption is that paid posters are only online virtual agents created by some normal individuals.

The averaging process mentioned above is also a basic approach usually used in non-Bayesian methods. A large amount of literature has studied how misinformation affects social learning in two canonical models: Bayesian and non-Bayesian learning. In these models, agents try to learn the truth by exchanging opinions with each other and learning from the messages received from the news. Non-Bayesian agents naively update beliefs by repeatedly taking weighted averages of their neighbors’ opinions [42]. In contrast, based on an underlying model of the reality, Bayesian agents are presumed to update their beliefs optimally (from a statistical perspective). Bayesian approaches require agents to have a reliable understanding of the world for assigning priors and to take others’ strategies into account [43]. Although these requirements make issues such as indoctrination and the spread of misinformation nearly impossible, they are still quite challenging [44]. Therefore, besides aggregating the opinions of the neighbors in a non-Bayesian way, we assume that an agent also computes their posterior beliefs by applying a simplified Bayesian analysis conditional on the messages received about the truth.

Furthermore, we introduce another type of epistemically irresponsible agent: agents who hide their honest opinions and focus on disseminating misinformation and who ignore the messages received and follow the crowd mindlessly.

The simulations illustrate that in an environment with only online selective neighbors, misinforming is more successful with broader confidence intervals. Having offline neighbors contributes to being cautious of misinformation, while employing Bayesian analysis helps in discovering the truth. Moreover, the agents, who are only willing to listen to the majority, whatever the truth, unwittingly help to bring about the success of misinformation attempts, and they themselves are, of course, misled to a greater extent.

We next provide a brief outline of the rest of this paper. In Section 2, we present an opinion update model with bounded confidence intervals and heterogeneous agents. Section 3 discusses the simulations and analyses. In Section 4, we conclude the paper.

## 2. Model

### 2.1. The Original BC Model

There is a true state of the world θ (whose exact nature we do not specify here). For simplicity, we assume that the opinion concerning θ can be expressed by a real number. Labeled by i∈V={1,2,…,n}, *n* agents interact with each other in a small social network at discrete points in time. Agent *i*’s opinion concerning θ at time *t* is denoted by $\mu_{i,t}\in [0,1]$. The two endpoints of that closed interval represent two extremely opposite attitudes towards θ: $\mu_{i}$ = 1 when agent *i* considers θ to be true and $\mu_{i}$ = 0 when agent *i* is completely misinformed by misinformation and considers θ to be false. As for the real numbers between those two endpoints, agents with opinions $\mu_{i,t}\geq$ 0.5 are inclined to believe θ, whereas those with opinions $\mu_{i,t}<$ 0.5 are more attracted to misinformation. Therefore, $\mu_{i}$ can also be interpreted as the probability that agent *i* believes θ.

In the BC model, when agents update their opinions, they only take into account those agents who are within their bounded confidence interval, meaning that their opinions differ from the agents less than a certain confidence level $r_{i}$. The set of agents within agent *i*’s bounded confidence interval at time *t* is given by

$$S\left(i,\mu_{t}\right)=\left\{1\leq j\leq n\left|\left|\mu_{i,t}-\mu_{j,t}\right|\leq r_{i}\right.\right\}$$

Thus, agent *i*’s opinion concerning θ at time *t* + 1 is defined to be

(1) $$\mu_{i,t+1}={\left|S\left(i,\mu_{t}\right)\right|}^{-1}\sum_{j\in S\left(i,\mu_{t}\right)}\mu_{j,t}$$

where $\left|\cdot \right|$ denotes the number of elements.

### 2.2. The Opinion Update Model

**Figure 1:**
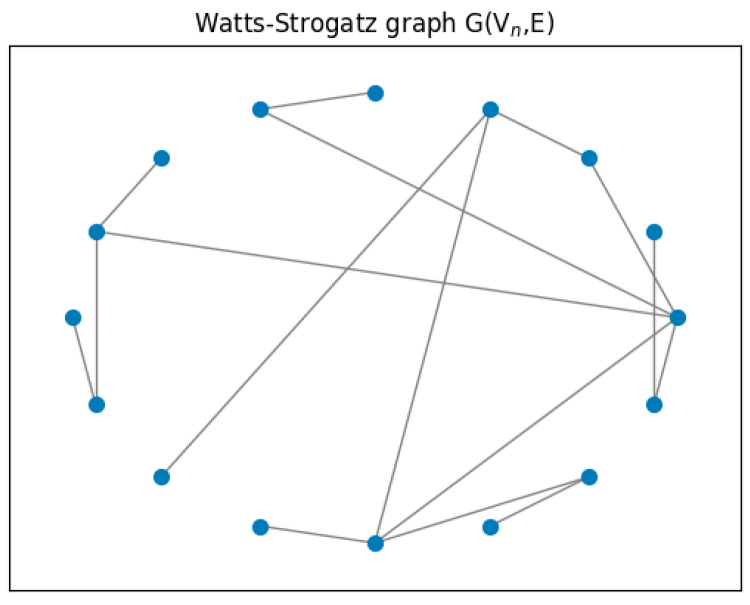
A Watts–Strogatz graph $G(V_{n},E)$ where each node in the set $V_{n}$ represents an agent and each undirected edge in the set *E* represents the connection between two agents.

Two communication patterns coexist in the social network: online and offline communication. When online, agents only communicate with those who fall within their bounded confidence intervals. In particular, anyone can be anyone’s neighbor regardless of their identities in real life. The aforementioned set of agents $S\left(i,\mu_{t}\right)$ will henceforth be called “online selective neighbors”. Offline neighbors are people whom an agent has to communicate with, although their opinions are far different from their own, and who may deeply influence them, such as their families and colleagues. Since we would like to discuss a network with ordinary non-media agents who have a similar number of neighbors, we represent this offline social network with a Watts–Strogatz small-world graph $G(V_{n},E)$, where each node in the set $V_{n}$ represents an agent and each undirected edge in the set *E* represents the connection between two agents (see Figure 1). Specifically, we say that agent *i* and agent *j* know each other if there is an undirected edge between *i* and *j*. Given that offline connections are relatively more stable, we assume that offline neighbors, denoted by $N\left(i,\mu_{t}\right)$, do not change in time.

To not complicate things too much, we assume that agent *i* equally weights all $j\in N\left(i,\mu_{t}\right)$:

(2) $${\mu_{i,t+1}}^{\prime }={\left|N\left(i,\mu_{t}\right)\right|}^{-1}\sum_{j\in N\left(i,\mu_{t}\right)}\mu_{j,t}.$$

Here, we add one more component to the opinion update process: “Bayesian analysis”. It has three key components: (i) priors, which are the agents’ existing beliefs about the underlying model of the world, and in this case, they are the agents’ opinions concerning θ at the previous time step, i.e., $\mu_{i,t}$; (ii) sources of information, which are the messages about θ that the agents receive from their surroundings or the news; and (iii) a method of information processing, i.e., Bayes’ rule. As a result, agent *i* forms a posterior opinion:

(3) $${\mu_{i,t+1}}^{\prime \prime }=\frac{\left(1-q\right)\mu_{i,t}}{\left(1-q\right)\mu_{i,t}+q\left(1-\mu_{i,t}\right)}.$$

where *q* denotes the probability that a message contains misinformation. In particular, *q* < 0.5.

Thus, by combining Equations (1)–(3), agent *i* updates their opinions according to:

(4) $$\mu_{i,t+1}=\alpha {\left|S\left(i,\mu_{t}\right)\right|}^{-1}\sum_{j\in S\left(i,\mu_{t}\right)}\mu_{j,t}+\beta {\left|N\left(i,\mu_{t}\right)\right|}^{-1}\sum_{j\in N\left(i,\mu_{t}\right)}\mu_{j,t}+\gamma \frac{\left(1-p\right)\mu_{i,t}}{\left(1-p\right)\mu_{i,t}+p\left(1-\mu_{i,t}\right)}$$

for weights *α*, *β*, and *γ* with *α* + *β* + *γ* = 1.

### 2.3. Typed Agents

**Figure 2:**
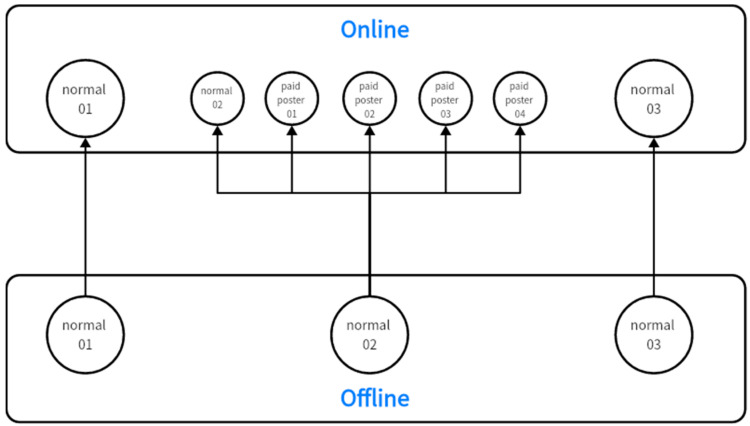
Besides being themself, normal 02 also creates 4 paid posters online.

The agents that populate Equation (4) will henceforth be called “normals”. Now, we introduce two new types of agents. The first is “paid posters”, who hide their honest opinions and focus on disseminating misinformation. They always stick to a fixed opinion (zero) and never update. It is worth noting that the behavior mentioned above is only applicable to online platforms. We can think of “paid posting” as a job. So, in one sense, paid posters are only online virtual agents created by some normals. In particular, there is not a one-to-one correspondence between normals and paid posters, meaning that one normal can create as many paid posters as she wants. As shown in Figure 2, normal 01 and normal 03 are only normals. They behave the same online and offline. Normal 02 accepts the job of “paid posting” and creates four virtual agents, i.e., paid posters 01 to 04. As a result, in addition to expressing their true opinions on online platforms, normal 02 also disseminates misinformation as paid posters.

The second new type of agent is “fence-sitters”, who ignore the messages about θ and follow the crowd mindlessly. In Section 2.1, we divided agents into two groups: agents with opinions $\mu_{i,t}\geq$ 0.5 that are inclined to believe θ and those with opinions $\mu_{i,t}<$ 0.5 that are more attracted to misinformation. Thus, 0.5 is a boundary between two sides, and the majority is the side with the larger number of agents. Thus, whatever the truth, fence-sitters are only willing to listen to the majority:

(5) $$\mu_{i,t+1}={\left|I\left(i,\mu_{t}\right)\right|}^{-1}\sum_{j\in I\left(i,\mu_{t}\right)}\mu_{j,t}$$

where $I\left(i,\mu_{t}\right)$ denotes the set of agents on the majority side ($\mu_{t}<$ 0.5 or $\mu_{t}\geq$ 0.5).

Then, the formal opinion update model is as follows:

(6) $$\mu_{i,t+1}=\left\{\begin{array}{l} \alpha {\left|S\left(i,\mu_{t}\right)\right|}^{-1}\sum_{j\in S\left(i,\mu_{t}\right)}\mu_{j,t}+\beta {\left|N\left(i,\mu_{t}\right)\right|}^{-1}\sum_{j\in N\left(i,\mu_{t}\right)}\mu_{j,t}\\ \begin{array}{ll} +\gamma \frac{\left(1-q\right)\mu_{i,t}}{\left(1-q\right)\mu_{i,t}+q\left(1-\mu_{i,t}\right)} & \mathrm{if}\;i\;\mathrm{is}\;a\;\mathrm{normal}\;\mathrm{agent}\\ {\left|I\left(i,\mu_{t}\right)\right|}^{-1}\sum_{j\in I\left(i,\mu_{t}\right)}\mu_{j,t} & \mathrm{if}\;i\;\mathrm{is}\;a\;\mathrm{fence-sitter}\\ 0 & \mathrm{if}\;i\;\mathrm{is}\;a\;\mathrm{paid}\;\mathrm{poster} \end{array} \end{array}.\right.$$

## 3. Simulations and Analyses

### 3.1. Online Selective Neighbors Only

Here, we consider the baseline case studied throughout the BC model literature. There are only selected neighbors and no offline neighbors or Bayesian analysis, that is, *α* = 1, *β* = 0, and *γ* = 0. The social network we utilized here and elsewhere include 50 normals whose initial opinions are drawn randomly from the interval [0, 1]. We assume that the confidence level is the same for all agents, i.e., $\forall i\in V,r_{i}=r$. Besides exploring the number of agents who are inclined to believe θ ($\mu_{i,t}\geq$ 0.5) or believe the misinformation ($\mu_{i,t}<$ 0.5), we paid more attention to the agents who have been completely misinformed. Theoretically, an agent with an opinion $\mu_{i}$ = 0 can be considered completely misinformed. However, to reduce the number of calculations, we say agent *i* completely believes the misinformation if she holds an opinion that almost equals zero, i.e., $\mu_{i}<10^{-2}$.

In the first experiment, we considered a variable number of paid posters and different values of *r*. In this setup, the questions that concerned us were how many normals would be completely misinformed and to what extent this depends on the number of paid posters and the value of *r* present in the social network.

**Figure 3:**
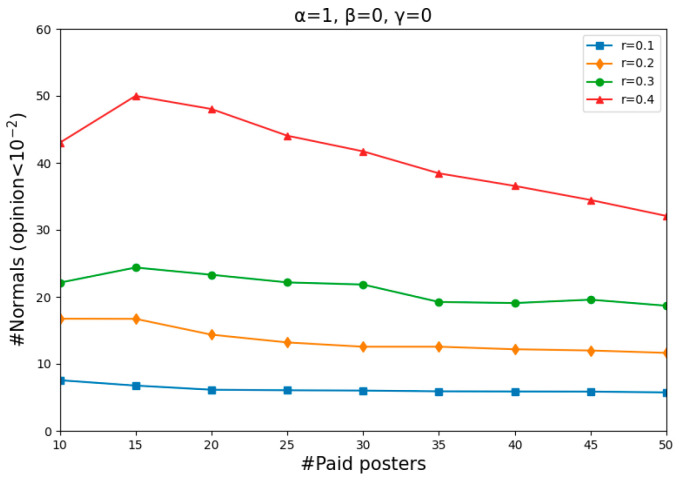
The number of normals whose opinions dropped below $10^{-2}$, with the number of paid posters increasing from 10 to 50 (in increments of 5) and *r* rising from 0.1 to 0.4 (in increments of 0.1). “#Paid posters” denotes the number of paid posters and “#Normals” denotes the number of normals.

Figure 3 shows, for each combination of the number of paid posters (from 10 to 50, in steps of 5) and the value of *r* (from 0.1 to 0.4, in steps of 0.1), the number of normals whose opinions have dropped below $10^{-2}$ in two simulations. For each of these nine steps of four different values of *r*, we repeat the simulation 50 times, starting with a different random start distribution each time. Each simulation was carried out until the dynamics became stable (stable means that for all agents *i*, $\left|\mu_{i,t+1}-\mu_{i,t}\right|\leq 10^{-5}$).

As shown in Figure 3, how many normals become entirely misinformed, on average, mainly depends on the willingness to count others as their peers. Specifically, the larger the value of *r*, the more successful the misinforming was. However, the results are largely insensitive to the number of paid posters when *r* is no more than 0.3 as there was not much difference in the number of normals who became entirely misinformed when there were 10 and 50 paid posters.

**Figure 4:**
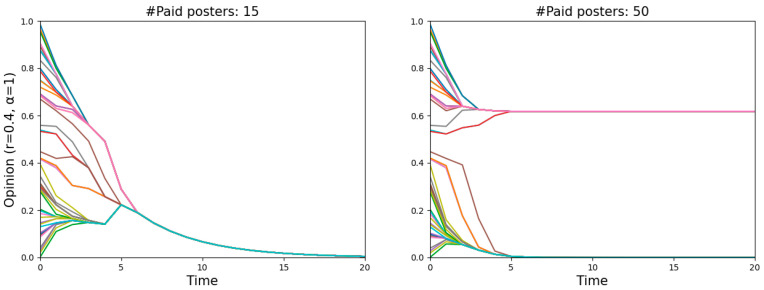
Illustration of why the misinforming is more successful with fewer paid posters than with more of them.

An obvious decline occurred with an increase in the number of paid posters when *r* = 0.4, meaning that the misinforming is more successful with fewer paid posters than with more of them, even though one might have expected to find the opposite. Such a puzzling phenomenon is actually quite understandable. Two single runs shown in Figure 4 illustrate the dynamics of *r* = 0.4 in more detail, one featuring 15 paid posters (left panel) and the other 50 (right panel). Since the starting distribution is not uniform, less populated areas, namely “gaps”, occur on the vertical lines (*t* = 0). The more paid posters there are, the harder they pull normals to zero, and the more likely a divergence occurs when there is a “gap”. In that case, the normals above the “gap” will never believe the misinformation. In contrast, fewer paid posters with a relatively weaker pull may attract all normals step by step and eventually convince them.

### 3.2. Adding Offline Neighbors and Bayesian Analysis

**Figure 5:**
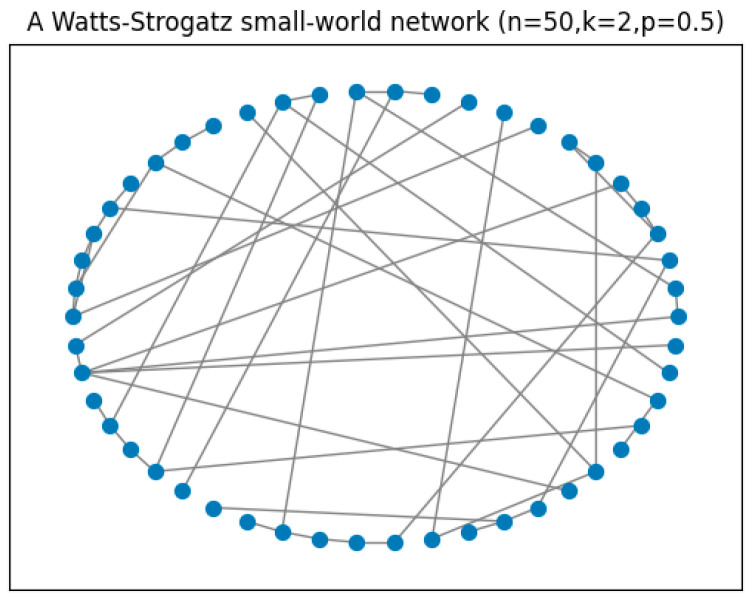
An example of a Watts–Strogatz small-world network with *n* = 50, *k* = 2, and *p* = 0.5.

Now, let us look at a more interesting setting: *α* = 0.5 and *β* = 0.5, indicating that normals update their opinions considering not only online selective neighbors but also offline neighbors. We assume a Watts–Strogatz small-world network as the offline network. Since the social network we present is a small one, we started with a ring lattice of 50 vertices with two edges per vertex, and each edge is then rewired at random with a probability of 0.5. Figure 5 gives an example of such a network.

**Figure 6:**
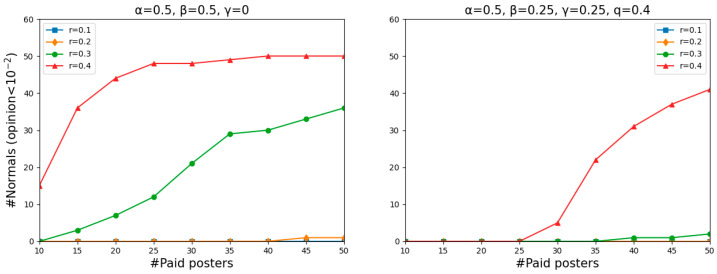
The number of normals, on average, whose opinions have dropped below $10^{-2}$ after adding offline neighbors (**left** panel) and further adding Bayesian analysis (**right** panel).

What Figure 3 and the left panel of Figure 6 have in common is that the larger the value of *r*, the more successful the misinforming was. As can be seen in the left panel of Figure 6, when *r* = 0.1 and *r* = 0.2, even though there were many paid posters, almost no one completely believed the misinformation. Because offline neighbors are usually families and colleagues who hide their virtual features in real life and communicate with each other more sincerely, normals will receive much less deliberate misinformation. That is to say, offline communication enhances the normals’ abilities to beware of misinformation when normals are relatively closed-minded. However, when *r* = 0.3 and *r* = 0.4, the opposite situation occurs. The two ascending curves mean that the misinforming was more successful with more paid posters than with fewer of them. This is because the “gaps” that divided the normals into two camps in Figure 3 did not matter anymore here. Agent *i*’s offline neighbors, whose opinions might differ significantly from agent *i*’s, can pull them to any side of the gap. Thus, more paid posters have a higher chance of pulling normals to “opinion zero”.

**Figure 7:**
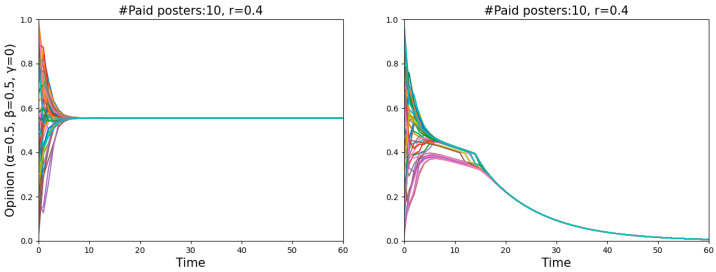
Two runs with identical parameter settings illustrate that for different starting distributions, normals sometimes will, and sometimes will not, become entirely misinformed.

We found that all normals can always reach a consensus after adding offline neighbors, signifying that either all normals or none became entirely misinformed (see Figure 7). Since the numbers shown in Figure 6 are averaged over 50 simulations (starting with a different random start distribution each time), many agents’ opinions dropping below $10^{-2}$ can be interpreted as a high probability of all normals being completely misled.

The right panel of Figure 6 shows the results after further adding a Bayesian analysis. Recall that the *q* is the probability that a message contains misinformation. We only consider the case where there is much misinformation and set *q* to be 0.4. Agents are more capable of being cautious of misinformation, where almost no one completely believes the misinformation even when *r* increases to 0.3. When *r* = 0.4, only a few normals were entirely misinformed when there were fewer paid posters. With the number of paid posters increasing from 30, the number of normals being pulled to the opposite of the truth moved upward rapidly. The changes in numbers may at first appear sudden, but in fact, they are easy to explain. The combination of a large *q* and a few paid posters indicates that a small number of agents are sending the same messages repeatedly. When there is rampant misinformation but a handful of people sending it, normals can employ their sophisticated reasoning abilities to infer from this paradox that some agents are spreading rumors and choose not to believe. When there are as many misleading messages as paid posters, normals will relax their vigilance to stand on the side of the misinformation that more and more people are disseminating because of the herding effect. As can be seen, agents, even with the Bayesian analysis, are much easier to be misled when there are many paid posters and a large amount of misinformation.

**Figure 8:**
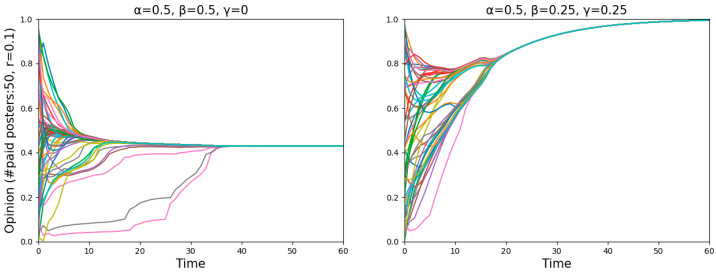
Comparison of two runs with identical parameter settings after adding offline neighbors (**left** panel) and further adding Bayesian analysis (**right** panel).

Focusing on the point “#paid posters: 50, *r* = 0.1”, the results in the two panels of Figure 6 are the same. However, on closer inspection, as seen in Figure 8, agents are near the dividing line between believing and disbelieving the misinformation in the left panel, and agents reach a consensus to believe the truth in the right panel. To put it differently, having offline neighbors contributes to being cautious of misinformation, while employing a Bayesian analysis helps in discovering the truth.

### 3.3. Adding Fence-Sitters

From this point on, we will only discuss the results simulated by the formal opinion update model in Section 2.3 (Equation (6)), which adds offline neighbors and a Bayesian analysis to the original BC model.

**Figure 9:**
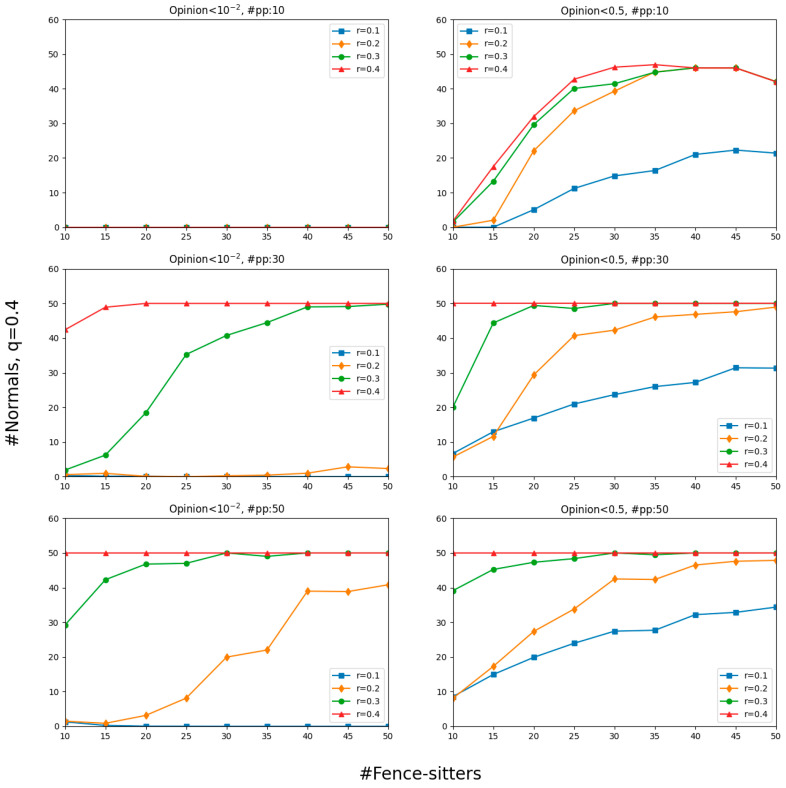
Under conditions of varying numbers of paid posters (**top** row: 10; **middle** row: 30; and **bottom** row: 50), the normals believe the misinformation to different degrees. The **left** column shows the results for the number of normal with opinions dropping below $10^{-2}$ and the **right** column shows the results for opinions less than 0.5. Results are averages over 50 simulations per number of fence-sitters and value of *r* combination. “#pp” is an abbreviation of “#paid posters”.

Until now, we have focused on social networks that consist of normals and paid posters. In this section, we consider the opinion dynamics with the addition of fence-sitters, who ignore the messages about θ and follow the crowd mindlessly. More exactly, we adopt six settings whose results are visualized in Figure 9: the top row with 10 paid posters, the middle row with 30, and the bottom row with 50. Each row contains two parts: the left column shows the results for opinions that dropped below $10^{-2}$ and the right column shows the results for opinions that were less than 0.5. To reduce complexity, we assumed that, for every fence-sitter, the number of the elements in their set of neighbors was 20, which comes from rounding the average of the numbers of all normals’ online selective neighbors at the staring points in 20 simulations (*r* = 0.2). The question we were interested in is what effect the presence of different numbers of fence-sitters has on the number of normals who have been misinformed.

As mentioned before, normals are not easily misled by misinformation when there are a few paid posters. However, many normals were misinformed with the gradual increase in the number of fence-sitters. In other words, the rising lines in Figure 9 indicate that the more fence-sitters there are, the more normals will lower their opinions to less than 0.5. In particular, the number of misguided normals with 50 fence-sitters surpassed that without fence-sitters—by far in most cases. That number, however, appears to be insensitive to the number of paid posters when there were more than 30: the risk brought about by “#pp: 50” was only slightly greater than that brought about by “#pp: 30”.

No matter how many fence-sitters there were and how liberal the normals were in counting others as their peers, no normals completely believed the misinformation (see upper left panel of Figure 9). Would this signify that the fence-sitters here were ineffective? The answer is no. The upper right panel of Figure 9 shows that more and more normals were pulled to the side of misinformation with a gradual increase in the number of fence-sitters. They begin to doubt the truth and gravitate towards the misinformation. The comparison between the two columns in Figure 9 also suggests that fence-sitters made more efforts to attract normals to the wrong side than to make them thoroughly misinformed.

**Figure 10:**
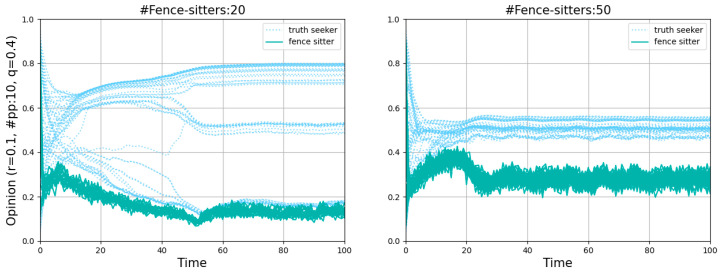
Comparison of opinion dynamics with 20 and 50 fence-sitters where there are a few paid posters and a small value of *r*.

Figure 10 shows the points “#fence-sitters:20, *r* = 0.1” and “#fence-sitters:50, *r* = 0.1” in the first row of Figure 9 in more detail. The fence-sitters’ opinions moved downward rapidly because of the existence of a few paid posters. The sudden increase in the number of agents who were inclined to believe the misinformation reinforced the fence-sitters’ conviction that they had made the correct choice. Vacillating between other fence-sitters’ opinions and zero, they never converged. Because the fence-sitters, as unwitting accomplices of paid posters, could appear in any normal agent’s set of offline neighbors, some normals may find the misinformation attractive and lower their values of opinions. Consequently, the presence of fence-sitters had an obvious negative effect on the normals. It only took a few fence-sitters to make the normals who have already reached a consensus on the truth appear indecisive about believing it or not.

Fence-sitters vacillated between other fence-sitters’ opinions and zero, and their opinions stayed between 0 and 0.5. Their exact position between 0 and 0.5 depended on the values of other parameters. The more misinformation there was, the more successful the paid posters were with the help of the fence-sitters, and the more the opinions of normals moved downward. Once the opinions of normals declined to below 0.5, the fence-sitters were then pulled up to be near the normals. Then, normals maintained their opinion, and the fence-sitters continued to vacillate between the normals and zero. Consequently, except for very few normals whose opinions were the nearest to zero at the very beginning, all of them were further from zero than the fence-sitters were.

As for the more general lesson we have learned from Figure 9, fence-sitters unwittingly help paid posters to bring about the success of misinformation attempts, but not to the point of making normals completely misinformed. Fence-sitters themselves are, of course, misled and nearer to zero than normals are.

## 4. Conclusions

Our project started from the assumption that the widespread misinformation in social networks, disseminated by ill-disposed agents, can mislead agents. Since rampant misinformation has posed significant threats to all areas of people’s lives, there is a practical interest in examining how and to what extent agents are misinformed by misinformation. As long as we understand where our greatest vulnerabilities lie when communicating with others, we are able to take measures to protect ourselves against epistemic manipulation.

With this in mind, we revisited the model, which combines Bayesian and non-Bayesian learning, and assumed the network topology to be a Watts–Strogatz small-world network. Therefore, we first proposed an extension of the model in which neighbors are divided into two parts: online selective neighbors whose opinions differ from their own but not by more than a certain confidence level, and offline neighbors whom an agent has to communicate with even though their opinions are far different from their own. In a further step, we introduced two new types of agents: first, paid posters, who only exist online, always stick to a fixed opinion (zero), and never update; second, fence-sitters, who are only willing to listen to the majority, regardless of the truth.

In an environment with only selective neighbors, the misinforming is more successful with broader confidence intervals. Having offline neighbors contributes to being cautious of misinformation, while employing a Bayesian analysis helps in discovering the truth. However, a Bayesian analysis does not work when there are many paid posters sending misinformation. Moreover, fence-sitters unwittingly help paid posters to bring about the success of misinformation attempts, but not to the point of making normals completely misinformed. Fence-sitters themselves are, of course, misled and nearer to zero than normals are.

We find that agents on the majority side serve as stochastic exogenous signals to make fence-sitters carry out random jumps inside the whole opinion space [45]. Influenced by those agents, fence-sitters are always vacillating about whether to believe the misinformation. Therefore, exploring more interesting phenomena induced by stochastic exogenous signals is left to be a future project.

Our research is subject to some limitations. Since this is a simulation study, we hope to conduct an empirical study based on real-world data to calibrate the model in the future. Furthermore, we represented the offline social network with a Watts–Strogatz small-world graph, which is inappropriate for a large social network since its degree distribution is not heavy-tailed. An extension of this work should consider scale-free networks.

## Data Availability

No new data were created or analyzed in this study.
